# Lipidomic Analysis of Serum from High Fat Diet Induced Obese Mice

**DOI:** 10.3390/ijms15022991

**Published:** 2014-02-20

**Authors:** Kristina Eisinger, Gerhard Liebisch, Gerd Schmitz, Charalampos Aslanidis, Sabrina Krautbauer, Christa Buechler

**Affiliations:** 1 Department of Internal Medicine I, Regensburg University Hospital, 93042 Regensburg, Germany; E-Mails: kristina.eisinger@klinik.uni-regensburg.de (K.E.); sabrina.krautbauer@klinik.uni-regensburg.de (S.K.); 2 Institute for Clinical Chemistry and Laboratory Medicine, Regensburg University Hospital, 93042 Regensburg, Germany; E-Mails: gerhard.liebisch@klinik.uni-regensburg.de (G.L.); gerd.schmitz@klinik.uni-regensburg.de (G.S.); charalampos.aslanidis@klinik.uni-regensburg.de (C.A.)

**Keywords:** phospholipids, lysophosphatidylcholine, lipidomic profiling, obesity, serum

## Abstract

Lipid metabolites regulate fatty acid and glucose homeostasis. The intention of the current study is to identify circulating lipid species, which are altered in rodent obesity and strongly correlate with the classically measured metabolites glucose, triglycerides, and cholesterol. Mice fed a high fat diet (HFD) for 14 weeks have increased body weight and fasting glucose. Serum triglycerides are not altered, while cholesterol tends to be increased. Accordingly, major cholesteryl ester (CE) species and free cholesterol are not significantly raised in obesity while minor metabolites, including CE 20:3 and CE 18:3, are increased or reduced, respectively. Distinct sphingomyelin (SM) species are elevated while ceramides are not raised. Phosphatidylinositol (PI) species, including PI 34:1, are raised while others are decreased. PI 34:1 strongly correlates with fasting glucose and proinsulin levels. Phosphatidylcholine (PC) 26:0, 40:2, and 40:5, which are induced in obesity, correlate with cholesterol. PC 38:4 and PC 40:6 are also raised in fat fed mice and positively correlate with fasting glucose. Lysophosphatidylcholine (LPC) species are also changed in obesity and the already shown reduction of LPC 16:1 has been confirmed. LPC 22:4, which is increased, correlates with serum cholesterol. The data indicate that circulating levels of various lipid species are changed in the obesity model studied and some of them are strongly associated with classically measured metabolites.

## 1. Introduction

The prevalence of obesity has dramatically increased over the past few decades [1,2]. Obesity is a primary risk factor for metabolic diseases, including type 2 diabetes, non-alcoholic fatty liver disease and cardiovascular disease, and presents a serious public health problem [3–5].

The mechanisms linking obesity to metabolic diseases are not precisely known. There is considerable evidence that impaired lipid metabolism plays a major role therein [1,3,4]. Recently developed lipidomic techniques demonstrate a high complexity of the plasma lipidome [6]. These methods are used to identify new lipid biomarkers associated with obesity and type 2 diabetes which may be relevant in pathophysiology, diagnosis, and therapy.

Lipid profiling in monozygotic twins reveals higher concentrations of lysophosphatidylcholine (LPC) and lower levels of ether phospholipids in serum of the obese twins [7]. Changes in these lipid species are associated with insulin resistance, independent of genetic factors [7]. Higher concentrations of LPC 14:0 and LPC 18:0 in overweight/obese men have been described in a second study, while LPC 18:1 is found reduced [8]. LPC 18:2 and sphingomyelin 16:1 in serum are inversely related to type 2 diabetes risk [9].

In plasma of mice, fed a high fat diet (HFD) for 12 weeks, LPC species are even decreased and most of them decline already after one week of HFD. Further, sphingomyelin, ceramide, and hexosylceramide levels are raised [10]. Most of the LPC species analyzed are also found reduced in a second study using mice fed a HFD for 10 weeks. LPC 17:0, 18:0, and 18:3 are, nevertheless, significantly induced. Total phosphatidylcholine (PC) concentration is increased about three-fold in obesity [11], whereas, choline and phosphorylcholine are reduced in serum of diet-induced obese rats [12].

Most studies have shown that ceramides are increased in rodent obesity [10,13,14], while decreased level of ceramide 24:1 has also been described [15]. In patients, ceramide levels are induced in obesity [16,17] and associated with markers of insulin sensitivity [17]. Holland *et al.* have proven that inhibition of ceramide synthesis by the serine palmitoyltransferase inhibitor myriocin improves obesity-associated insulin resistance [18]. Further, blockage of acid sphingomyelinase lowers HFD mediated ceramide generation and body weight gain [13]. Phosphatidylcholine 18:0/18:1 is a diurnal serum lipid in which temporal changes are dysregulated in obesity. Treatment of db/db mice with PC 18:0/18:1 improves lipid and glucose metabolism [19]. These data further confirm a strong link between lipid- and glucose homeostasis. Although most studies demonstrate increased serum ceramide in obesity, data on further lipid metabolites are inconsistent. In the current study, various lipid species have been measured in serum of male mice fed a standard chow or a high fat diet for 14 weeks.

## 2. Results

### 2.1. Metabolic Profile of Fat Fed Mice

**Figure 1.**
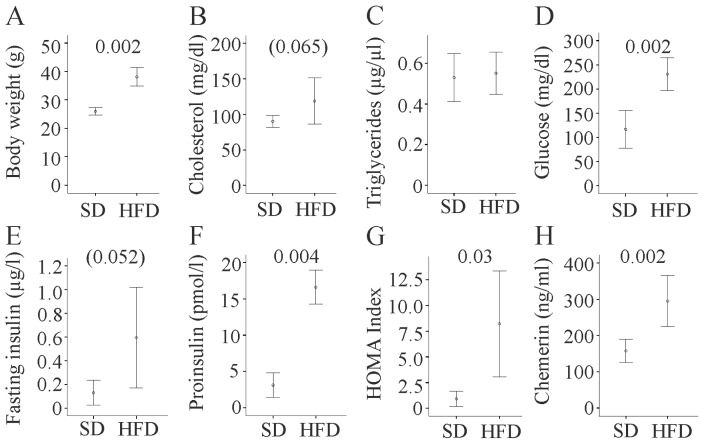
Metabolic parameters of C57BL/6 mice fed a standard chow (SD) or a high fat diet (HFD) for 14 weeks. (**A**) Body weight; (**B**) Total cholesterol; (**C**) Triglycerides; (**D**) Fasting glucose; (**E**) Fasting insulin; (**F**) Proinsulin; (**G**) HOMA Index; and (**H**) Chemerin were measured in the serum of these animals. Numbers in the figure indicate *p*-values, *p*-values indicating a trend are given in brackets.

The six mice on a high fat diet (HFD) had a body weight of 39.3 (32.5–41.3) g, which was significantly higher compared to the six mice on a standard diet (SD) with 25.8 (23.9–27.5) g (Figure 1A). Total cholesterol tended to be elevated, while triglycerides in serum were not raised (Figure 1B,C). Fat fed mice displayed higher fasting blood glucose, tended to have increased fasting insulin, had raised proinsulin levels, and increased Homeostasis model assessment (HOMA) index (Figure 1D–G). The adipokine chemerin was markedly increased in serum of HFD mice (Figure 1H) as described [20,21].

### 2.2. Cholesterol Species

**Figure 2.**
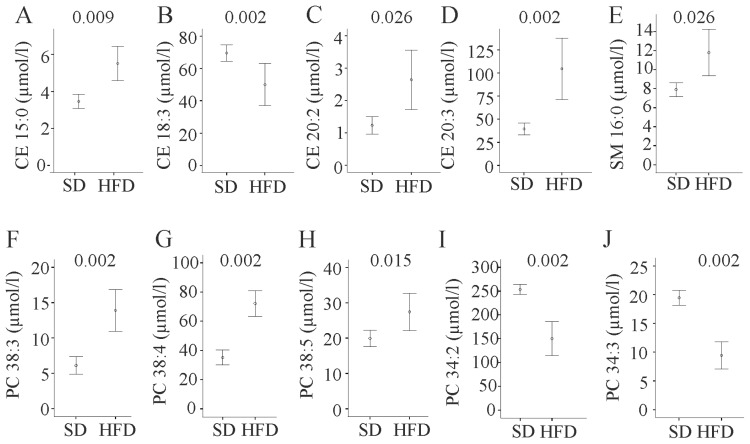
Cholesteryl ester (CE), sphingomyelin (SM), and phosphatidylcholine (PC) species in serum of mice fed a standard chow (SD) or high fat diet (HFD) for 14 weeks. (**A**) CE 15:0; (**B**) CE 18:3; (**C**) CE 20:2; (**D**) CE 20:3; (**E**) SM 16:0; (**F**) PC 38:3; (**G**) PC 38:4; (**H**) PC 38:5; (**I**) PC 34:2; and (**J**) PC 34:3 were measured in the serum of these animals. Numbers in the figure indicate *p*-values.

Total cholesterol measured with a commercially available assay (Figure 1B) and mass spectrometry were highly correlated (*r* = 0.958, *p* < 0.001) and levels tended to be higher (*p* = 0.065) in serum of fat fed animals. Free cholesterol levels showed a similar trend (Table S1A). Concentrations of total saturated, monounsaturated (MUFA), and polyunsaturated (PUFA) cholesteryl ester (CE) species measured were similar in serum of SD and HFD fed mice (data not shown). Ratios of CE 18:1 to CE 18:2 (the preferred fatty acid of tissue acyl-CoA cholesterol acyltransferase (ACAT) and serum lecithin cholesterol acyltransferase (LCAT), respectively [22]) were significantly (*p* = 0.004) increased in HFD (data not shown). Analysis of single CEs revealed raised CE 15:0, CE 20:2 and CE 20:3, while CE 16:1 and CE 18:3 were decreased in HFD (Figure 2A–D, Table S1A).

### 2.3. Sphingomyelin and Ceramides

Total sphingomyelin (SM) was 39.3 (26.4–47.3) μmol/L in serum of HFD animals and 28.9 (24.8–33.3) μmol/L in SD fed mice and was significantly higher in the first group (*p* = 0.041). Here, total saturated and total monounsaturated fatty acid (MUFA) species but not polyunsaturated (PUFA) SM were raised (*p* = 0.041 for both comparisons). Elevated levels of these SM classes are explained by higher SM 16:0 (Figure 2E) and 18:0 (*p* = 0.009), and higher SM 16:1 (*p* = 0.041), SM 18:1 (*p* = 0.026), and SM 22:1 (*p* = 0.041) in serum of HFD fed mice (Table S1B). Ceramides were similarly abundant in serum of SD and HFD fed mice (Table S1C).

### 2.4. Phosphatidylcholine

Total, MUFA, PUFA, and saturated phosphatidylcholine (PC) species were not altered (data not shown). PC 26:0, 36:1, 38:3, 38:4, 38:5, 40:2, 40:5, and 40:6 were significantly increased. PC 34:2, 34:3, and 36:0 were significantly decreased (Figure 2F–J, Table S1D).

### 2.5. Lysophosphatidylcholine

Total lysophosphatidylcholine (LPC), MUFA, and saturated LPC species were not changed upon HFD, while PUFA LPC species were significantly reduced (*p* = 0.015). LPC species altered in serum of HFD animals are listed in Table 1. Kim *et al.* and Barber *et al.* [10,11] already analyzed LPC species in serum of SD and HFD animals and comparison of their and current findings showed that LPC 16:1 was the only species which was consistently decreased in all the fat fed mice models studied. Other LPC species were not congruently altered (Table 1).

### 2.6. Phosphatidylinositol

**Figure 3.**
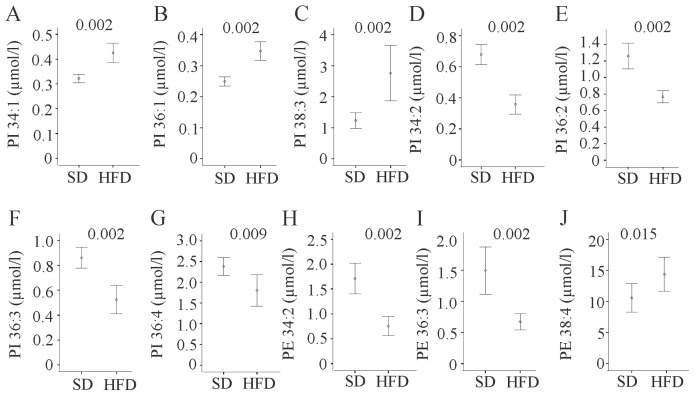
Phosphatidylinositol (PI) and phosphatidylethanolamine (PE) species in serum of mice fed a standard chow (SD) or high fat diet (HFD) for 14 weeks. (**A**) PI 34:1; (**B**) PI 36:1; (**C**) PI 38:3; (**D**) PI 34:2; (**E**) PI 36:2; (**F**) PI 36:3; (**G**) PI 36:4; (**H**) PE 34:2; (**I**) PE 36:3; and (**J**) PE 38:4 were measured in the serum of these animals. Numbers in the figure indicate *p*-values.

Total and PUFA phosphatidylinositol (PI) tended to be increased (*p* = 0.065 for both comparisons) and MUFA PIs were significantly higher (*p* = 0.002) in obesity. PI 34:1 (Figure 3A), 36:1 (Figure 3B) and 38:3 (Figure 3C) were raised in HFD. PI 34:2 (Figure 3D), 36:2 (Figure 3E), 36:3 (Figure 3F) and 36:4 (Figure 3G) were decreased. Data of all PI species measured are summarized in Table S1E.

### 2.7. Phosphatidylethanolamine

Total phosphatidylethanolamine (PE), MUFA, PUFA, and saturated PE were not altered (data not shown). PE 34:2 and PE 36:3 were diminished and PE 38:4 was raised in HFD (Figure 3H–J). Data of all PE species measured are listed in Table S1F.

### 2.8. Body Weight Independent Correlations

**Figure 4.**
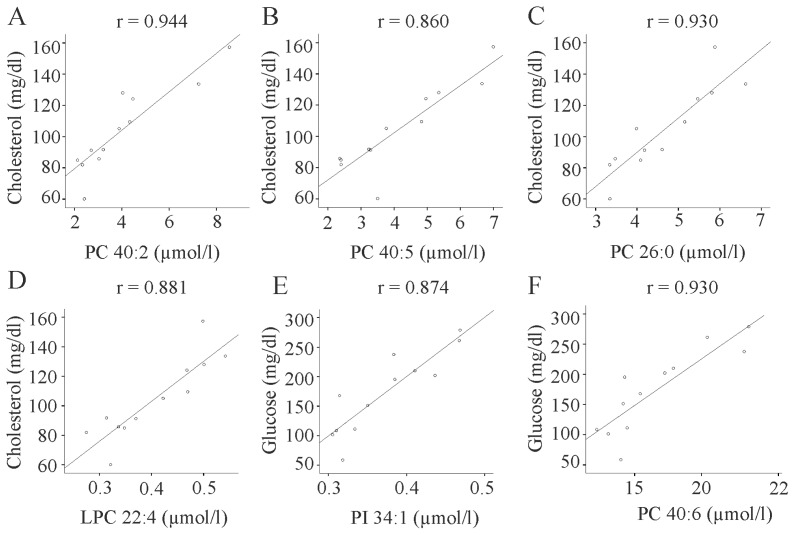
Correlation of lipid species with systemic cholesterol and fasting glucose (**A**) Correlation of PC 40:2 with cholesterol; (**B**) Correlation of PC 40:5 with cholesterol; (**C**) Correlation of PC 26:0 with cholesterol; (**D**) Correlation of LPC 22:4 with cholesterol; (**E**) Correlation of PI 34:1 with fasting glucose; (**F**) Correlation of PC 40:6 with fasting glucose.

Correlations of the different lipids and total serum cholesterol, triglycerides, insulin, proinsulin, glucose, and HOMA index were calculated and highly significant associations (*p* ≤ 0.001), which were still significant after adjusting for body weight are shown. PC 26:0, 40:2, 40:5 and LPC 22:4 positively correlated with total cholesterol (Figure 4A–D). After correcting for body weight, respective *p*-values for the lipids above were 0.001, 0.001, <0.001 and 0.002. PI 34:1, PC 38:4 (*r* = 0.846, *p* = 0.001), and PC 40:6 were positively associated with fasting glucose (Figure 4E,F and data not shown). After correcting for body weight the respective *p*-values were 0.010, 0.033, and 0.009. PI 34:1 also positively correlated with proinsulin (*r* = 0.845, *p* = 0.001, and *p* = 0.012, after adjusting for body weight). There were no significant correlations with fasting insulin, triglycerides, and HOMA index (data not shown).

## 3. Discussion

Nutrient oversupply and physical inactivity cause overweight/obesity which is a major factor in the pathogenesis of metabolic diseases [1–5]. Lipids are a highly diverse group of molecules regarding their structure and function [6,23,24]. Purpose of the current study is to get insight into the changes of serum lipids in obesity. Lipidomic analysis was performed in fat fed mice, a commonly used model to study obesity [10,11,18]. These mice gain more weight, have increased fasting glucose and HOMA index while total triglycerides are not elevated. Total cholesterol tends to be higher and, subsequently, most of the cholesteryl ester species measured are either unchanged or increased. CE 16:1 and CE 18:3 are the only derivatives which are reduced. Ratios of CE 18:1 to CE 18:2, the preferred fatty acids of tissue ACAT and serum LCAT, respectively [22] is significantly increased in HFD. CE 18:1 tends to be higher suggesting a trend to higher ACAT activity in obesity in line with the literature [25].

Analysis of total lipid concentrations revealed that only sphingomyelin is modestly but significantly increased in mice fed a HFD. This is partly caused by higher levels of SM 18:0 and 18:1, which have already been described to be raised in fat fed mice [10] and ob/ob mice [26]. Ceramide which is raised in serum of ob/ob mice and diet-induced obese animals [10,26] is, however, not induced in the serum of HFD fed mice studied herein suggesting that body weight gain is not associated with higher serum ceramides in general.

Lysophosphatidylcholine species have already been measured in rodent and human obesity [7,8,10,11]. Comparison of current data and results from Kim *et al.* and Barber *et al.* who used fat fed male C57BL/6 and male C57BL/6J mice, respectively, revealed that LPC 16:1 is the only species which is consistently decreased in these three animal models [10,11]. LPC 18:0 is raised in the mice studied by Kim *et al.* and Barber *et al.* [10,11] and, at least, tends to be induced in the serum of fat fed animals used in the present study. LPC 22:4 has only been analyzed in the current model and is increased. Interestingly, this lipid positively correlates with serum cholesterol.

These data suggest that most of the obesity-associated alterations in LPC species identified so far are specifically affected in the respective models studied but are not universally changed in mice chronically fed high fat diets. Considering that even levels of serum triglycerides and total cholesterol are not consistently induced in mice fed high fat diets [10–12,27] it is not surprising that most of the lipid species analyzed in these different mouse models are not uniformly changed.

Concentrations and composition of fatty acids vary in different diets. Dietary fatty acids exert multiple functions, partly by activation of specific transcription factors [28], and this may influence levels of distinct lipid species. The role of palmitate in ceramide metabolism has been studied in detail [23]. Other constituents in the diets also affect cellular function and, most likely, the lipid profile [29,30]. Therefore, type of diet, duration of feeding a high fat diet and time of day and/or time of fasting before collecting serum may affect lipidomic profile independent of obesity [10,19,31]. Gender also affects lipid levels [32,33] but only male mice have been enrolled in the three studies compared herein [10,11].

Most of the phosphatidylethanolamine species analyzed are not altered in obesity in accordance with published findings [10]. PE 38:4 is increased in the HFD model used herein and in the mice studied by Barber *et al.* [10].

To our knowledge phosphatidylinositol species have not been measured in rodent models of obesity so far. Some of the PI species analyzed are increased or decreased in serum of HFD fed animals. PI 34:1 representing about 1% to 2% of the PI species determined is increased in obesity. Of note, this lipid strongly correlates with fasting serum glucose and proinsulin levels. Of the various phosphatidylcholine species analyzed, only PC40:6 is also induced in the study by Barber *et al.* [10]. PC 40:6 positively correlates with serum glucose levels suggesting an association of this lipid with glucose homeostasis. PC 38:4 is also associated with fasting glucose concentrations and is found increased in the current animal model and unchanged in a recent study [10]. PC 26:0, 40:2, and 40:5, which are all elevated in the serum of fat fed mice strongly and positively correlate with serum cholesterol. Whether these associations indicate a functional relationship or co-regulation of these lipids needs further investigation.

Of note, none of the lipid species changed in obesity shows a strong correlation with HOMA index as marker of insulin resistance. Furthermore, no strong correlations with serum triglyceride levels have been identified arguing against a prominent role of a single lipid species in insulin resistance and serum triglyceride levels.

This comprehensive lipidomic analysis shows that sphingomyelins, glycerophospholipids, and cholesteryl ester species are altered in obesity at least in the rodent model studied herein. For our data the same limitations apply, as in all cross-sectional studies describing associations and not causal relationships. Thus, the pathways affected in obesity and the relevance of the identified biomarkers in obesity are still unknown. Furthermore, there is no established method to discriminate the effects related to the high fat diet and obesity. It is also important to note that serum of male mice has been analyzed.

A major challenge for the future is the characterization of the individual functions of the various lipid species circulating in blood. Where possible current data have been compared to results of additional studies and only a few of these lipids, namely LPC 16:1, SM 18:0, SM 18:1, PE 38:4, and PC 40:6 are concordantly changed [10,11]. Therefore, these lipids are at least good candidates to further study their role in obesity using large cohorts of human samples to identify if any differences in the plasma lipid profile exist between obese and non-obese individuals.

## 4. Experimental Section

### 4.1. Materials

Triglyceride concentrations were measured using GPO-PAP microtest (purchased from Roche, Mannheim, Germany) and total cholesterol in serum was determined by using an assay from Diaglobal (Berlin, Germany). Proinsulin and insulin were determined by the appropriate ELISAs from Mercodia (Uppsala, Sweden). Glucose was measured by QuantiChrom Glucose Assay Kit from Biotrend (Köln, Germany). The Homeostasis model assessment (HOMA) index was calculated using the formula: [fasting glucose (mmol/L) × fasting insulin (mU/L)]/22.5.

### 4.2. Animal Model

Mice were ordered from The Jackson Laboratory (Bar Harbor, ME, USA) and housed in a 21 ± 1 °C controlled room under a 12 h light-dark cycle. Animals had free access to food and water and were housed with 3 to 5 mice per cage. Blood was drawn after fasting overnight. Rising concentrations of CO_2_ were used to produce loss of consciousness followed by cervical dislocation. Procedures were approved by the University of Regensburg Laboratory Animal Committee and complied with the German Law on Animal Protection and the Institute for Laboratory Animal Research Guide for the Care and Use of Laboratory Animals, 1999.

Fourteen week old male C57BL/6 mice were kept on a high fat diet (HFD) or standard chow (SD) for 14 weeks. Feed composition of these diets can be downloaded from the homepage of this company (Ssniff, Soest, Germany)

Gross energy of SD (ssniff^®^ EF acc. D12450B (I) mod.) was 17.8 MJ/kg, 70% of kJ were from carbohydrate, 20% from protein and 10% from fat. Gross energy of HFD (ssniff^®^ EF R/M acc. D12451 (II) mod.) was 22.1 MJ/kg, 35% of kJ were from carbohydrate, 20% from protein and 45% from fat (Ssniff, Soest, Germany). Fatty acid composition and cholesterol content of these diets are listed in Table S2. For technical reasons data of insulin, proinsulin, and HOMA index are shown for 11 mice, all other data are given for 12 mice.

### 4.3. Quantification of Lipids

Lipids were quantified by direct flow injection electrospray ionization tandem mass spectrometry (ESI-MS/MS) in positive ion mode using the analytical setup and strategy described previously [34]. A precursor ion of *m*/*z* 184 was used for phosphatidylcholine (PC) [34]. A neutral loss of 141 and 277 Da were used for phosphatidylethanolamine (PE) and phosphatidylinositol (PI) [35], respectively. Sphingosine-based ceramides (Cer) were analyzed using a fragment ion of *m*/*z* 264 [36]. Free cholesterol (FC) and cholesteryl ester (CE) were quantified using a fragment ion of *m*/*z* 369 after selective derivatization of FC [37]. Lipid species were annotated according to the recently published proposal for shorthand notation of lipid structures that are derived from mass spectrometry [38]. Glycerophospholipid annotation is based on the assumption of even numbered carbon chains only. SM species annotation is based on the assumption that a sphingoid base d18:1 is present. In case the fatty acid composition was not determined, annotation represents the total number of carbons and double bonds. For example, PC 36:4 comprises species like PC 16:0/20:4 or 18:2/18:2.

### 4.4. Statistical Analysis

Data are presented as mean values ± standard deviation. Statistical differences were analyzed by two-tailed Mann-Whitney *U* Test (SPSS Statistics 19.0 program, IBM, Leibniz Rechenzentrum, München. Germany) and a value of *p* < 0.05 was regarded as significant. Spearman correlations (IBM SPSS Statistics 19.0 program) were calculated. Correlations with *p* ≤ 0.001 which were still significant (*p* < 0.05) after adjusting for body weight are shown.

## 5. Conclusions

The lipid species LPC 16:1, SM 18:0, SM 18:1, PE 38:4, and PC 40:6 seem to have a role in rodent obesity.

## Supplementary Information



## Figures and Tables

**Table 1.** Lipid species measured in serum of mice fed a standard chow (SD) or high fat diet (HFD) for 14 weeks.

Lipid	SD	Std. dev.	HFD	Std. dev.	*p*-value	Regulation	Regulation Kim *et al.*	Regulation Barber *et al.*
LPC 15:0	0.48	0.05	0.55	0.10	0.093	-	↓	↓
LPC 16:1	9.36	0.67	5.51	0.94	**0.002**	↓	↓	↓
						
LPC 16:0	69.84	4.11	60.02	13.01	0.180	-	↓	↓
LPC 18:3	1.80	0.11	0.90	0.16	**0.002**	↓	↑	n.d.
LPC 18:2	39.02	2.24	18.04	3.14	**0.002**	↓	↓	-
LPC 18:1	82.73	5.60	84.19	16.24	0.310	-	↓	↓
LPC 18:0	26.76	2.60	39.35	8.13	0.065	-	↑	↑
LPC 20:5	0.48	0.09	0.57	0.08	0.180	-	↓	↓
LPC 20:4	21.15	2.60	26.00	2.83	**0.026**	↑	↓	↑
						
LPC 20:3	2.21	0.44	4.35	0.69	**0.002**	↑	n.d.	-
LPC 20:0	2.10	0.25	1.24	0.26	**0.002**	↓	n.d.	-
LPC 22:6	6.65	0.58	6.93	1.22	0.485	-	n.d.	-
LPC 22:5	0.81	0.10	1.11	0.31	0.065	-	n.d.	n.d.
LPC 22:4	0.34	0.05	0.47	0.07	**0.026**	↑	n.d.	n.d.
LPC 22:0	3.81	0.35	5.23	1.50	0.065	-	n.d.	n.d.

The mean values (in μM) ± standard deviation (Std. dev.) are listed. Regulation indicates increased (↑)/decreased (↓)/unchanged (−) levels in serum of HFD fed mice compared to SD fed animals. Significant *p*-values are shown in bold letters. Regulation described by Kim *et al.* [11] and Barber *et al.* [10] is shown for comparison. Lipid species consistently regulated in the three studies are highlighted in dark grey. Differentially regulated lipid species measured in at least two studies are highlighted with light grey. (Not determined, n.d.).
