# Supplementary material for: Lipidomic Analysis of Serum from High Fat Diet Induced Obese Mice
Source: Int J Mol Sci. 2014 Feb 20;15(2):2991–3002. doi: 10.3390/ijms15022991 (PMC3958895; doi:10.3390/ijms15022991)
Supplement: Supplementary file 1 [file ijms-15-02991-s001.pdf]

Supplementary Information

Table S1. Lipid species measured in serum of mice fed a standard chow (SD) or high fat diet (HFD) for 14 weeks.

A. Free cholesterol (FC) and cholesteryl ester (CE) species.

Lipid	SD	Std. dev.	HFD	Std. dev.	*p*-value	Regulation	Regulation. Barber *et al.*
FC	304.2	30.6	401.9	92.0	0.065	-	n.d.
CE 14:0	8.63	0.88	10.55	2.12	0.093	-	n.d.
CE 15:0	3.46	0.35	5.51	0.84	**0.009**	↑	n.d.
CE 16:1	172.27	18.55	133.70	31.61	**0.026**	↓	n.d.
CE 16:0	54.99	6.18	55.45	10.88	0.818	-	n.d.
CE 18:3	69.42	4.58	49.96	11.89	**0.002**	↓	n.d.
CE 18:2	697.94	39.62	705.84	172.64	0.132	-	n.d.
CE 18:1	123.05	16.00	171.44	37.77	0.065	-	n.d.
CE 18:0	5.19	0.72	5.46	0.54	0.485	-	n.d.
CE 20:5	24.12	3.13	28.52	6.65	0.093	-	n.d.
CE 20:4	1870.87	164.98	3088.34	835.48	0.065	-	n.d.
CE 20:3	39.35	5.87	104.51	30.35	**0.002**	↑	n.d.
CE 20:2	1.24	0.25	2.64	0.84	**0.026**	↑	n.d.
CE 22:6	262.13	19.74	328.05	98.82	0.065	-	n.d.
CE 22:5	13.80	0.89	22.77	7.64	0.065	-	n.d.
CE 22:4	2.31	0.35	2.90	0.73	0.240	-	n.d.

B. Sphingomyelin (SM) species (assignment is based on the assumption that a sphingoid base d18:1 is present).

Lipid	SD	Std. dev.	HFD	Std. dev.	*p*-value	Regulation	Regulation. Barber *et al.*
SM 14:0	0.46	0.25	0.68	0.14	0.132	-	↑
SM 15:0	0.64	0.14	0.78	0.24	0.699	-	↑
SM 16:1	1.25	0.10	1.48	0.19	**0.041**	↑	-
SM 16:0	7.90	0.67	11.80	2.23	**0.026**	↑	n.d.
SM 18:1	0.43	0.23	0.87	0.25	**0.026**	↑	↑
SM 18:0	1.01	0.24	1.89	0.44	**0.009**	↑	↑
SM 22:1	1.33	0.78	2.27	0.51	**0.041**	↑	-
SM 22:0	2.51	1.02	3.67	0.97	0.132	-	-
SM 23:1	0.77	0.24	0.85	0.14	0.485	-	n.d.
SM 23:0	1.09	0.28	1.21	0.42	0.485	-	n.d.
SM 24:2	2.15	0.47	1.64	0.52	0.132	-	-
SM 24:1	6.98	0.40	8.11	1.66	0.132	-	↓
SM 24:0	1.60	0.16	1.66	0.55	0.589	-	-

C. Ceramide (Cer) species.

Lipid	SD	Std. dev.	HFD	Std. dev.	*p*-value	Regulation	Regulation. Barber *et al.*
Cer d18:1/16:0	0.40	0.05	0.38	0.08	0.699	-	-
Cer d18:1/18:0	0.22	0.03	0.22	0.06	0.394	-	↑
Cer d18:1/20:0	0.25	0.05	0.28	0.05	0.394	-	↑
Cer d18:1/22:0	0.63	0.18	0.57	0.13	0.937	-	↑
Cer d18:1/23:0	0.63	0.12	0.59	0.10	0.699	-	n.d.
Cer d18:1/24:1	1.70	0.29	1.30	0.33	0.093	-	-
Cer d18:1/24:0	0.92	0.09	0.82	0.20	0.394	-	-

D. Phosphatidylcholine (PC) species (assignment is based on the assumption that two acyl bonds are present).

Lipid	SD	Std. dev.	HFD	Std. dev.	*p*-value	Regulation	Regulation. Barber *et al.*
PC 26:0	3.95	0.43	5.38	1.01	**0.041**	↑	n.d.
PC 32:2	1.51	0.76	1.49	0.88	0.818	-	-
PC 32:1	10.83	1.02	9.11	2.35	0.132	-	-
PC 32:0	12.99	1.42	13.08	2.68	0.937	-	-
PC 34:4	0.73	0.23	0.42	0.34	0.180	-	n.d.
PC 34:3	19.46	1.17	9.41	2.16	**0.002**	↓	-
PC 34:2	253.38	9.67	149.97	32.50	**0.002**	↓	-
PC 34:1	182.35	7.69	244.31	73.55	0.065	-	-
PC 34:0	2.00	1.10	3.27	1.60	0.310	-	-
PC 36:5	4.04	0.87	3.82	0.55	1.000	-	-
PC 36:4	83.92	7.12	104.02	16.62	0.065	-	↑
PC 36:3	40.25	2.25	36.35	5.90	0.310	-	↑
PC 36:2	76.18	7.38	77.12	9.46	0.818	-	-
PC 36:1	20.50	0.95	37.11	6.43	**0.002**	↑	-
PC 36:0	10.17	1.86	6.29	1.47	**0.002**	↓	n.d.
PC 38:7	2.31	0.35	2.01	0.82	0.699	-	n.d.
PC 38:6	42.59	2.98	44.96	10.21	0.394	-	-
PC 38:5	19.88	2.09	27.40	4.81	**0.015**	↑	↓
PC 38:4	35.16	4.59	72.13	8.08	**0.002**	↑	-
PC 38:3	6.11	1.13	13.89	2.69	**0.002**	↑	-
PC 38:2	5.25	0.59	6.06	2.11	0.394	-	-
PC 38:1	3.64	0.22	4.20	0.98	0.132	-	n.d.
PC 38:0	3.50	0.59	2.95	0.87	0.310	-	n.d.
PC 40:7	9.67	1.26	9.58	1.67	1.000	-	-
PC 40:6	13.86	1.03	19.43	3.32	**0.009**	↑	↑
PC 40:5	2.91	0.55	5.38	1.17	**0.004**	↑	-
PC 40:4	3.60	0.36	4.72	1.63	0.065	-	n.d.
PC 40:3	2.17	0.68	1.92	1.33	0.699	-	n.d.
PC 40:2	2.89	0.58	5.18	2.07	**0.026**	↑	n.d.
PC 40:1	0.93	0.63	1.61	0.72	0.240	-	n.d.
PC 40:0	4.05	1.15	4.74	2.26	0.699	-	n.d.
PC 42:0	1.60	0.51	2.95	1.13	0.093	-	n.d.

E. Phosphatidylinositol (PI) species (assignment is based on the assumption that two acyl bonds are present).

Lipid	SD	Std. dev.	HFD	Std. dev.	*p*-value	Regulation	Regulation. Barber *et al.*
PI 34:2	0.68	0.06	0.36	0.06	**0.002**	↓	n.d.
PI 34:1	0.32	0.02	0.43	0.03	**0.002**	↑	n.d.
PI 36:4	2.38	0.20	1.80	0.35	**0.009**	↓	n.d.
PI 36:3	0.86	0.08	0.52	0.10	**0.002**	↓	n.d.
PI 36:2	1.26	0.14	0.77	0.07	**0.002**	↓	n.d.
PI 36:1	0.25	0.01	0.35	0.03	**0.002**	↑	n.d.
PI 38:6	0.21	0.02	0.18	0.03	0.132	-	n.d.
PI 38:5	2.03	0.25	2.04	0.33	0.937	-	n.d.
PI 38:4	17.45	1.02	25.16	6.23	0.065	-	n.d.
PI 38:3	1.23	0.23	2.76	0.81	**0.002**	↑	n.d.
PI 40:6	0.27	0.03	0.24	0.03	0.180	-	n.d.
PI 40:5	0.14	0.05	0.17	0.03	0.180	-	n.d.
PI 40:4	0.17	0.04	0.20	0.06	0.937	-	n.d.

F. Phosphatidylethanolamine (PE) species (assignment is based on the assumption that two acyl bonds are present).

Lipid	SD	Std. dev.	HFD	Std. dev.	*p*-value	Regulation	Regulation. Barber *et al.*
PE 34:2	1.71	0.28	0.75	0.17	**0.002**	↓	-
PE 36:4	2.48	0.47	2.42	0.30	0.485	-	-
PE 36:3	1.50	0.35	0.67	0.12	**0.002**	↓	-
PE 36:2	2.03	0.27	1.77	0.37	0.589	-	-
PE 36:1	0.57	0.20	0.77	0.18	0.132	-	↑
PE 38:6	13.49	3.82	12.26	3.39	0.589	-	-
PE 38:5	8.47	2.32	9.33	1.65	0.310	-	-
PE 38:4	10.55	2.09	14.38	2.51	**0.015**	↑	↑
PE 38:2	1.50	0.38	1.54	0.26	0.699	-	-
PE 38:1	1.24	0.16	0.92	0.66	0.937	-	-
PE 40:6	3.50	1.10	3.55	0.80	0.937	-	-
PE 40:5	1.88	0.67	1.58	0.26	0.589	-	n.d.

The mean values (in µM) ± standard deviation (Std. dev.) are listed. Regulation indicates increased (↑)/decreased (↓)/unaltered (-) levels in serum of HFD fed mice compared to SD fed animals. Significant *p*-values are shown in bold letters. Regulation of the lipid species measured in the current study was compared to the data of Barber *et al.* [10]. Lipid species consistently regulated in the current study and in the mice analyzed by Barber *et al.* are highlighted in dark grey. Differentially regulated lipid species in the two studies are highlighted with light grey. (Not determined, n.d.).

Table S2. Lipid composition of the diets.

Lipid	SD	HFD
**Fatty acids**	**[%]**	**[%]**
C 8:0	-	-
C10:0	-	-
C12:0	0.01	0.02
C14:0	0.04	0.69
C16:0	0.66	5.39
C16:1	0.06	0.52
C17:0	-	0.25
C18:0	0.33	3.75
C18:1	1.31	8.17
C18:2	1.44	1.84
C18:3	0.17	0.25
C20:0	0.01	0.03
C20:1	-	0.01
C20:4	0.03	0.05
C20:5	-	-
C22:6	-	-
**Cholesterol**	**[mg/kg]**	**[mg/kg]**
	14	194

Data of fatty acids are given as % (*w*/*w*) of the chow as provided by the manufacturer. Standard chow (SD); high fat diet (HFD).

© 2014 by the authors; licensee MDPI, Basel, Switzerland. This article is an open access article distributed under the terms and conditions of the Creative Commons Attribution license (http://creativecommons.org/licenses/by/3.0/).
